# Erysipelas Complicated with Acute Exudative Pericarditis

**DOI:** 10.3390/medicina56110571

**Published:** 2020-10-29

**Authors:** Akvilė Gečaitė, Aušra Vainalavičiūtė, Daiva Emilija Rekienė, Laima Jankauskienė, Albinas Naudžiūnas

**Affiliations:** 1 Department of Medicine, Faculty of Medicine, Lithuanian University of Health Sciences, 44307 Kaunas, Lithuania; akvilegecaite15@gmail.com; 2 Department of Medicine, Clinic of Internal Diseases, Lithuanian University of Health Sciences, 47144 Kaunas, Lithuania; daiva.rekiene@gmail.com (D.E.R.); Laima.Jankauskiene@lsmuni.lt (L.J.); albinas.naudziunas@lsmuni.lt (A.N.)

**Keywords:** erysipelas, acute exudative pericarditis, pericardium, pericardial fluid, arrhythmia

## Abstract

Erysipelas is a common skin infection of the upper dermis. Its most common complications are local; these include abscess formation, skin necrosis, etc. In the present article, we introduce a case of a 75-year-old patient with erysipelas of the face complicated with acute exudative pericarditis. The patient came to Kaunas Clinical Hospital complaining of extreme fatigue and fever, oedema of the left side of the face, and erythema typical for erysipelas. The patient also felt sternum and epigastric pain, especially during breathing, and dyspnoea. Heart work was rhythmic 100 bpm; blood pressure was 142/70 mmHg. Pericardial friction rub was heard over the left sternal border. There were no alterations in other systems. In the electrocardiogram, concave ST segment elevation in leads II, III, and aVF was identified. In addition, during hospitalisation, the patient experienced atrial fibrillation paroxysm, which was treated with amiodarone intravenously. The blood test showed C-reactive protein: 286 mg/L; white blood cells: 20 × 10^9^/L; troponin I was within the normal range. During echocardiography, pericardial fluid in pericardial cavity was identified. As no changes in troponin I were observed, according to the ST segment elevation, the woman was diagnosed with erysipelas of the left side of the face complicated with acute exudative pericarditis. Antibacterial treatment of cephalosporins was administered. After the treatment, C-reactive protein decreased to 27.8 mg/L; whereas, in the electrocardiogram, the return of the ST segment to the isoline was observed, and pericardial fluid resorbed from the pericardial cavity. To the best of the authors’ knowledge, this case is a rare combination of erysipelas complicated with acute exudative pericarditis.

## 1. Introduction

Erysipelas tends to be a mild skin infection; yet, in a lot of cases, it still requires in-hospital treatment [1]. Local complications of erysipelas—haemorrhagic, bullous, abscessing, and necrotic lesions—are significantly more frequent than the systemic ones [2]. In our case, the patient developed a rare complication of erysipelas—pericarditis. 

Pericarditis is an inflammatory disease of the pericardium that can have infectious and non-infectious causes [3]. Pericarditis can be triggered by bacterial exposure, viruses, tuberculosis, drugs, trauma, parasitic, or neoplastic causes [4]. 

In this article, we describe a clinical case of a 75-year-old patient who was hospitalised because of erysipelas, which complicated with acute exudative pericarditis. In addition, we analyse and compare the case with the available data in the literature.

## 2. Case Presentation

**Figure 1:**
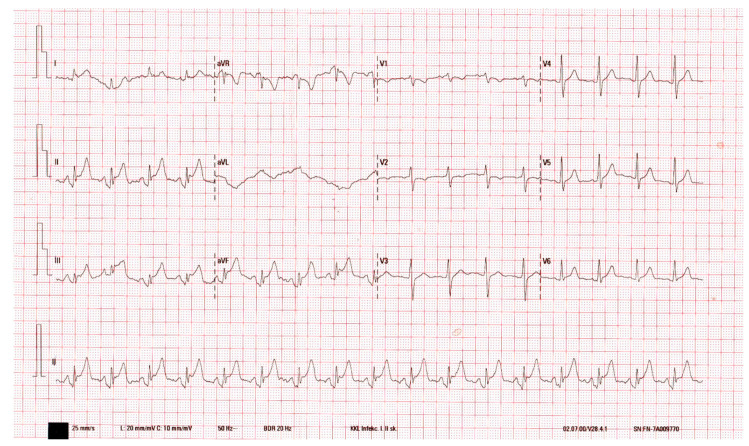
Day 2 of the disease. Sinus rhythm. Concave ST segment elevation in leads II, III, and aVF.

A 75-year-old female was admitted to the emergency room because of an oedema on the left side of her face, erythema, extreme fatigue, and fever. The patient had a temperature of up to 40 °C along with chills, but only on the first day of the hospitalisation. The patient also felt persistent chest pain behind the sternum and in the epigastric area, especially during breathing, as well as dyspnoea. During the examination, the heart activity was rhythmic—100 bpm—the blood pressure measured 142/70 mmHg. The respiration rate was 25 breaths per minute, SpO2—99%. There was an erythematous skin lesion with a sharply demarcated edge, haemorrhages, and hard, infiltrated skin on the left side of the face. Swollen left submandibular lymph nodes were palpable. During auscultation, pericardial friction rub was heard over the left sternal border, and breathing was vesicular. The woman had no comorbidities, allergies, nor had she been taking any medications regularly. In general, there were no notable alterations in any other systems. ECG showed 1.5 mm concave ST segment elevation in leads II, III, and aVF, which was observed for the first five days of hospitalisation (Figure 1).

Regarding laboratory tests, the following values were observed: CRP—286 mg/l; WBC—20 × 10^9^/L; troponin I—0.012 mIU/mL (norm < 0.034 mIU/mL); other laboratory blood test results are presented in the table below (Table 1). No bacterial growth was detected in the blood culture. The chest radiograph was performed, but it did not show any abnormalities; the QuantiFERON test for tuberculosis was negative. Echocardiography showed a moderate amount (1.8 cm) of pericardial fluid in pericardial cavity, without any signs of cardiac tamponade. The ejection fraction of the heart was 55%. During hospitalisation, no troponin I elevation was identified, and considering the pericardial fluid, the possibility of the acute coronary syndrome was not further considered. Therefore, our patient was not referred for coronary angiography. Other tests (CT, MRI, nuclear scintigraphy) were not performed because our patient refused to undergo them.

The woman was hospitalised to Kaunas Clinical Hospital with erysipelas of the face and its complication—acute exudative pericarditis. Antibacterial treatment was initiated—the patient was treated with ceftazidime 2 g × 3 intravenously for the first 5 days of the treatment (thus the total dose was 30 g). Then, due to a shortage of ceftazidime, cefotaxime 1 g × 2 was administered for the next 14 days (the total dose was 28 g). The patient was also treated with ibuprofeni 400 mg × 2 orally, metoprololi 50 mg × 1 on days 5–7 and 50 mg × 2 on days 8–19, ketonali 2 mL × 1 intramuscularly, and sol. Ringeri 500 mL intravenously. The management of pericardial effusion was conservative, with clinical and echocardiographic surveillance.

**Figure 2:**
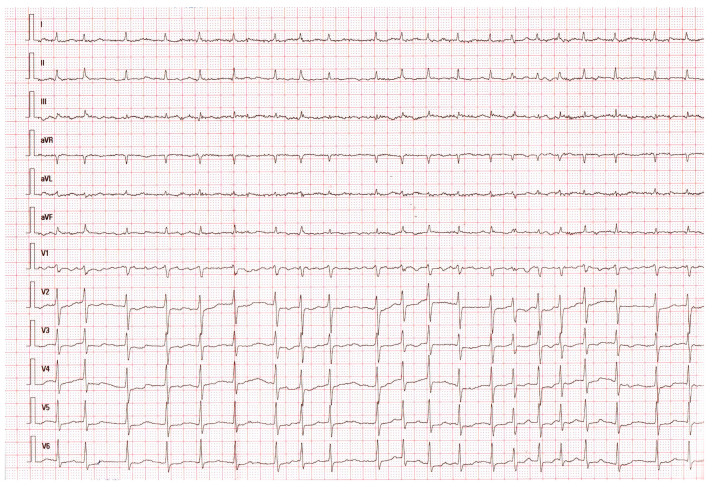
Day 13 of the disease. Atrial fibrillation.

In addition, during the hospitalisation (on hospitalisation days 6, 7, 8, and 13), the patient experienced recurrent atrial fibrillation (Figure 2) episodes, which were treated with intravenous amiodarone.

**Figure 3:**
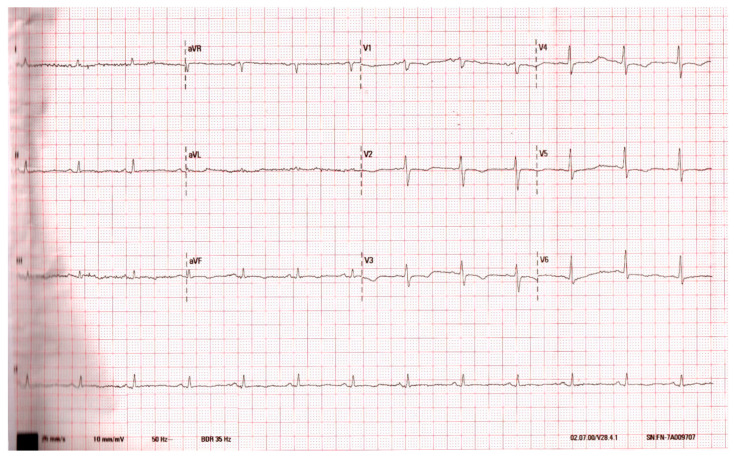
Day 15 of the disease. Sinus rhythm. ST segment returned to the isoline in leads II, III, and aVF.

After the treatment, the patient’s condition improved, erythema and oedema diminished, and inflammatory parameters decreased (CRP—27.8 mg/L). In the ECG, the return of the ST segment to the isoline was observed (Figure 3). Pericardial fluid disappeared, and the patient was released.

## 3. Discussion

Erysipelas is a skin infection characterised by an inflammatory reaction of the upper dermis, which extends into the superficial cutaneous lymphatics [5,6]. Usually, facial erysipelas is caused by group A streptococcus, while the infection of the lower extremities is more commonly caused by non-group A streptococcus [7]. Chronic medical conditions, such as cardiopulmonary or renal disease, diabetes, immunocompromise, and older age, are among the risk factors for bacterial skin and soft tissue infections [8]. In our case, the patient was in the risk group of an increased possibility of this infection due to the older age and gender. Erysipelas is described as an acute onset of local signs, which include progressing erythema, pain, and oedema. The lesion of the skin features clearly demarcated edges, which can help to differentiate erysipelas from other skin infections [6]. Our patient was presented with the above-mentioned symptoms specific to erysipelas; she was also suffering from fever of up to 40 °C. Major local complications of erysipelas include haemorrhagic, bullous, abscessing and necrotic lesions, and in some cases, myocarditis [2]. In our particular case, we observed an uncommon complication – acute pericarditis. 

Infectious causes of pericarditis are bacterial, viral, parasitic, and fungal, while non-infectious cases include drugs, trauma, neoplastic, metabolic, and autoimmune causes. In developing countries, tuberculosis is considered to be an important cause of pericarditis; however, in developed countries, most cases are idiopathic/viral [9]. In our particular case, acute pericarditis was caused by erysipelas. Pericardial effusion occurs in sixty percent of the patients with acute pericarditis [3]. In our case, during echocardiography, pericardial fluid was identified. Some authors claim that pericarditis can also cause atrial fibrillation [10]. During hospitalisation, our patient started to experience atrial fibrillation episodes, which may have been the result of acute pericarditis, as she had never experienced any serious arrhythmia before. 

Although the relation between erysipelas and acute myocarditis was previously described in a few cases [11], the relation between erysipelas and pericarditis is less common. This unusual association was mentioned in one retrospective cross-sectional study conducted at Hospital de Base, São José do Rio Preto, Brazil, with 219 women and 209 men who were hospitalised with suspected erysipelas. Out of the patients, 1.7% died, and the cause of the death of one patient was purulent pericarditis [12]. Nevertheless, the sample size of this study is relatively small, and the underlying conditions were mentioned as predisposing factors for death because of the complications of erysipelas. Patients with comorbidities have a weakened immune system and thus face a higher risk of developing complications similar to the ones presented in the above mentioned article, which results in an increased death rate. However, our patient did not have any comorbidities or allergies, she was not even taking any medications regularly. This allows us to conclude that she was in relatively good health before developing erysipelas. We assume that this clinical manifestation of erysipelas complicated with acute exudative pericarditis without any other underlying conditions is unique.

## 4. Conclusions

An uncommon clinical case was presented. Our patient was a 75-year-old female who was diagnosed with facial erysipelas complicated with acute exudative pericarditis. Despite this complication, the patient’s condition improved significantly after administration of cephalosporins. A few weeks later, the patient’s condition improved, and she was released home. In the course of hospitalisation, the patient also experienced recurrent atrial fibrillation episodes and was treated with amiodarone.

## Figures and Tables

**Table 1:** Laboratory test results.

On Day 1 of the Disease		On Day 5 of the Disease		On Day 13 of the Disease	
Tests	Result	Tests	Result	Tests	Result
CRP	**286.92 mg/L** 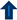	CRP	**242.0 mg/L** 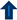	CRP	**47.2 mg/L** 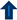
HGB	**125 g/L**	HGB	**110 g/L** 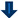	HGB	**116 g/L** 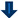
HCT	**38.4%**	HCT	**33.3%**	HCT	**33.4%**
WBC	**20.01 × 10^9^/L** 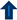	WBC	**15.0 × 10^9^/L** 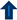	WBC	**5.7 × 10^9^/L**
PLT	**155 × 10^9^/L**	PLT	**328 × 10^9^/L**	PLT	**479 × 10^9^/L**
Potassium	**4.0 mmol/L**	Potassium	**4.7 mmol/L**	Potassium	**3.6 mmol/L**
